# Teaching ultrasound-guided peripheral venous catheter placement through immersive virtual reality: An explorative pilot study

**DOI:** 10.1097/MD.0000000000026394

**Published:** 2021-07-09

**Authors:** Nanna L. Andersen, Rune O. Jensen, Stefan Posth, Christian B. Laursen, Rasmus Jørgensen, Ole Graumann

**Affiliations:** a Department of Radiology, Odense University Hospital; b Research and Innovation Unit of Radiology, University of Southern Denmark; c Department of Emergency Medicine; d Department of Respiratory Medicine, Odense University Hospital; e Odense Respiratory Research Unit (ODIN), Department of Clinical Research, University of Southern Denmark, Denmark.

**Keywords:** education, immersive virtual reality, peripheral venous cannulation, ultrasound

## Abstract

**Introduction:**

Immersive virtual reality (IVR)-based training is gaining ground as an educational tool in healthcare. When combined with well-established educational methods, IVR can potentially increase competency and autonomy in ultrasound (US)-guided peripheral venous cannulation.

The aim of this study was to examine the impact of adding IVR training to a course in US-guided peripheral venous cannulation.

**Methods:**

Medical students (n = 19) from the University of Southern Denmark with no former standardized US education were recruited to voluntarily participate in a pilot study, designed as a randomized controlled trial. The primary outcome was the proportion of successful peripheral venous cannulations on a phantom. Secondary outcomes included the proportion of surface punctures on the phantom and procedure time. Participants received e-learning on the basic US before randomization to either IVR (n = 10) or no further training (n = 9). The additional IVR training comprised 10 virtual scenarios for US-guided peripheral venous catheter (PVC) placement. Students were subsequently evaluated in peripheral venous cannulation by a blinded assessor.

**Results:**

The proportion of successful peripheral venous cannulations was significantly higher in the IVR group (*P* ≤ .001). The proportions of successful cannulations were significantly higher in the IVR group compared to the control group for the 1st and 2nd PVC (*P* = .011, *P* = .023), but not for the 3rd PVC (*P* = .087). Similar results were found for the proportion of surface punctures (1st: *P* ≤ .001, 2nd: *P* = .001, and 3rd: *P* = .114). No significant differences in procedure times were found between the groups.

**Conclusion:**

This pilot study showed that adding an IVR-based training simulation to an existing e-learning curriculum significantly increased the learning efficacy of US-guided PVC placement for medical students.

## 1 Introduction

Mastering the skill of peripheral venous cannulation is essential across several medical specialties and professions.^[1,2]^ Peripheral venous catheters (PVC) are used for several medical interventions, including the intravenous administration of drugs, saline or glucose, and for blood transfusions.^[3]^

However, the procedure for peripheral venous cannulation can be complicated by numerous factors such as obesity, dehydration, hypovolemia, and hematologic diseases.^[4]^ Multiple attempts at cannulation may cause patients discomfort and increase the risk of complications such as phlebitis and subcutaneous infections.^[5,6]^ In some cases, clinicians are forced to use less optimal solutions (eg, central venous catheters or intraosseous access) for intravenous access.^[7]^

Ultrasound (US) guidance could increase the success rates of healthcare workers in placing PVCs. Using high-frequency US waves, US provides the healthcare worker with real-time images of the scanned tissue, including veins, tendons, and muscles.^[8–10]^ Studies have shown that US-guided PVC placement enables the visualization of non-visible or palpable veins in patients with difficult intravenous access, increases success rates and patient satisfaction, and requires less time to perform, and fewer vein punctures.^[2,11,12]^

Several studies on US-guided PVC placement training have reported positive results: a randomized crossover study by Vitto et al found a 100% success rate for medical students (n = 122) using US-guidance for PVC placement compared to a 56% success rate for traditional PVC placement after two 30-minute lectures on standard and US-guided peripheral venous cannulation, respectively.^[13]^ Furthermore, the average number of attempts to obtain venous access was significantly lower for the US-guided group (1.31 vs 2.16, *P* < .001). Similarly, McCarthy et al found a higher success rate for PVC placement in emergency department patients with moderate or difficult intravenous access when technicians used US guidance compared to a traditional landmark method (81.2% vs 71.4% and 81.6% vs 35.1%, respectively).^[12]^

US-guided peripheral venous cannulation may be useful for a high percentage of healthcare staff, but developing competency requires practice and supervision. Unfortunately, no consensus has been reached on either the best educational methods or the ideal setup for courses in US guidance.^[14–17]^

Virtual reality (VR)-based training is gaining ground as an educational tool in the medical field.^[18–20]^ In immersive VR (IVR), the user wears a headset and is placed in a simulated virtual environment, where either controllers or hand-tracking is used to create an interactive environment. The immersive setup provides a content-rich experience that can improve user engagement and thus, the training outcome.^[21]^ IVR can be used to practice procedures and develop practical competencies in safe and controlled environments.

In a cross-sectional study, Adhikari et al investigated simulation training and US-guided PVC placement and found that all participating emergency department nurses (n = 40) demonstrated competency in performing the procedure on a human model and a Blue Phantom model.^[22]^ After the training, 98% of the participants indicated that they preferred real-time US guidance over the static approach, and 92% agreed that the simulation training was adequate to learn the procedure. In a randomized controlled trial, İsmailoğlu et al investigated the effect of having nursing students practice intravenous catheterization skills using a virtual haptic device compared to video-assisted teaching and found no significant difference in post-test or self-confidence scores, but the students in the virtual simulator-group scored higher on psychomotor skills.^[23]^ These results suggest that simulation-based training could serve as an educational tool for US-guided PVC placement. Adding the immersive element of IVR to existing educational material could increase users’ competency and autonomy even further with regard to US-guided peripheral venous cannulation before the procedure is applied to patients.

The aim of the study was to examine the impact that adding IVR training to a course in US-guided peripheral venous cannulation for medical students had on the proportion of successful PVC placements, surface punctures, and procedure time. The primary outcome was the proportion of successful cannulations in the phantom. The secondary outcomes were procedure time for the US-guided PVC placement, divided into prescan and tip tracking times, and the proportion of surface punctures on the phantom correlated to the proportion of successful cannulations.

## 2 Methods

This double blinded, explorative pilot study was designed as a randomized controlled trial. Participants (n = 19) were medical students from the University of Southern Denmark (SDU) enrolled in the 5th to 11th semester (3rd to 6th year), 10 of which were enrolled in their 6th semester. Baseline characteristics for the participants are shown in Table 1.

**Table 1:** Baseline characteristics for the 2 groups.

	Intervention group (n = 10)	Control group (n = 9)
Sex		
Men	3	5
Women	7	4 (3)^∗^
Age in years		
21–25	6	8
26–30	4	0
31–35	0	1
Handedness		
Right	9	8
Left	1	1
Semester (from beginning)		
5th	1	1
6th	5	5
7th	0	1
8th	1	0
9th	1	1
10th	1	1
11th	1	0

∗ Data points for procedure time for the 3rd peripheral venous catheter were excluded for 1 participant in the control group because the participant did not reach this task within the time frame (15 minutes for 3) and could not be assessed in it.

Students were recruited to voluntarily participate through a written invitation shared on an internal communication platform for medical students. To meet the inclusion criterion, students had to have passed a practical exam in their 4th semester that included a short course in PVC placement. To minimize the risk of confounding, the students who had formerly received any standardized US education would be excluded. All participants were informed about the purpose of the study and they signed a consent form before the study began.

### 2.1 Randomization

Each participant picked an envelope containing a number: 1 for the IVR group or 2 for the control group. The allocation ratio for the groups was approximately 1:1, which resulted in 10 participants for the IVR group and 9 for the control group. The assessor who collected data was blinded, and all participants were instructed not to reveal to which group they had been assigned. All participants went through the learning material and the assessment described below on an individual basis.

### 2.2 Training videos

**Figure 1:**
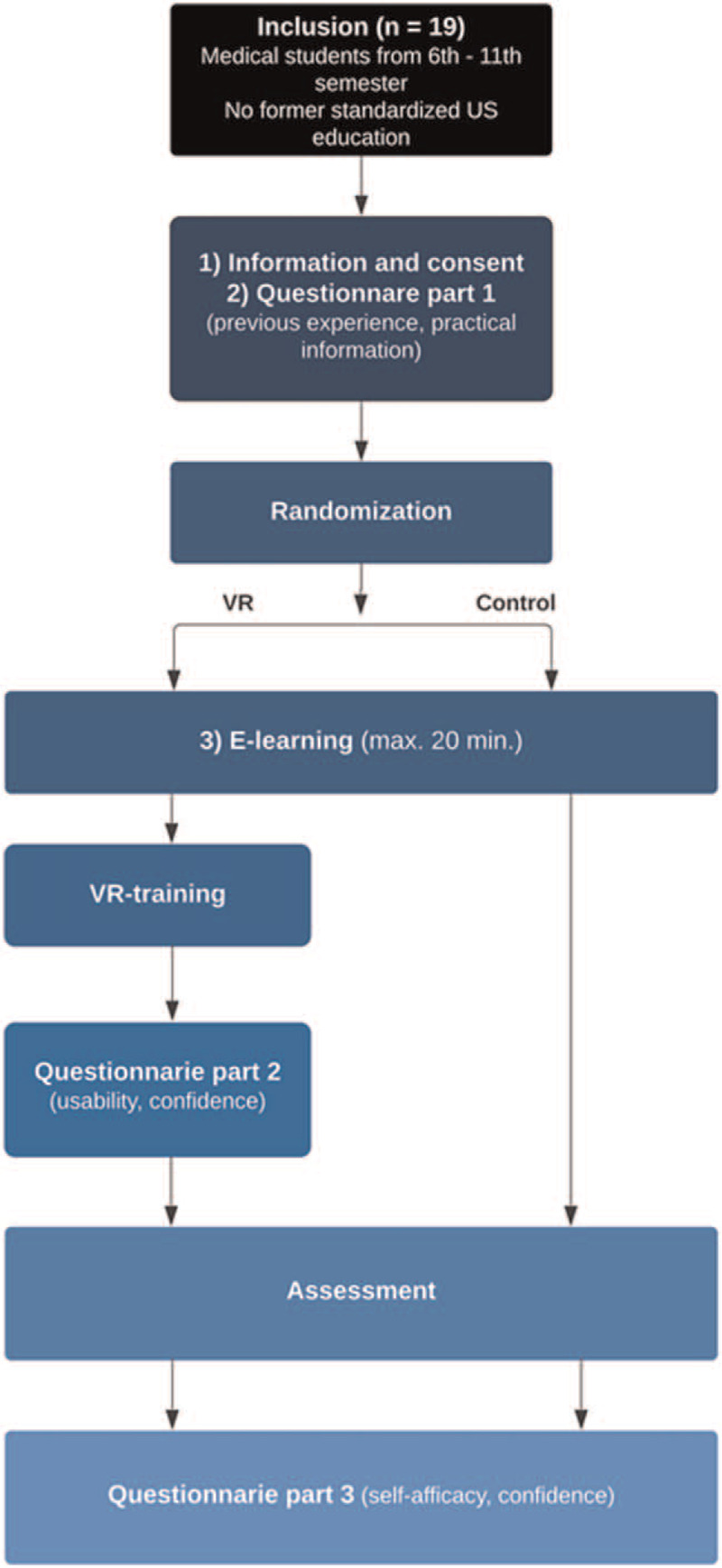
Flowchart of the US course. US = ultrasound, VR = virtual reality, USGIVA = Ultrasound Guided Intravenous Access.

To introduce US-guided PVC placement, all the participants individually watched 2 videos and read an accompanying text with images on a laptop (IBM) in a small, 3 × 3 meter classroom. The first video showed an instructor scanning the aorta of a patient while introducing the US probe, positioning of the patient, and optimization of the US image (5 minutes) and the accompanying text described basic knobology and the algorithm for PVC placement.^[24]^ The second video described and demonstrated how to perform US-guided PVC placement on a patient using the out-of-plane technique and tip tracking (3 minutes, 12 seconds).^[25]^ The participants in the control group received no further training on US-guided PVC placement and directly to the assessment (see below). The flow of the study is presented in Figure 1.

### 2.3 Intervention

After studying the material described above, the IVR group proceeded to IVR training one by one. Each participant wore a head-mounted device to access the simulation and interacted with it using 2 controllers. A VR-ready computer (specifications: i7-6800K, 64GB RAM, GTX 1080 8GB) was used to run the IVR simulator (HTC Vive System, Taipei, Taiwan; VR USGIVA 1.0 VitaSim, Odense, Denmark). The purpose of the simulation was to give the participant practice with tip tracking and thereby improve competency in this specific skill. The virtual environment consisted of a basic room containing only a US machine, a table, and a screen for instructions. Participants watched a short tutorial slideshow that introduced them to the controls, the task, and the tip tracking exercise.

The tactile aspect of PVC placement was simulated using 2 VR controllers and a physical table as a firm surface. One controller was used as a US transducer and the other to hold and operate the PVC. The participants were shown pictures of how to hold the controller in their dominant hand to obtain the best and most realistic usage when practicing PVC placement: The grip on the controller was pointed forward and the user held the controller in the base of the hand. This enabled the participant to pick up the virtual PVC at the tip of the controller to place the PVC.

The virtual PVC placement training included 10 short virtual scenarios lasting approximately 20 minutes in total. The aim of each scenario was to have the student place a PVC in a deep vein that was only visible using the US. The participants were instructed to make a cross-sectional scanning plane and use tip tracking. No phantoms were used in the simulation; however, a physical table was placed in front of the participants and its height was aligned with the virtual phantom which the participants scanned in IVR. This was done to simulate the physical feel of a firm, scannable surface. During scanning, a US image would appear on the virtual US machine and the participants would attempt to place the PVC inside the simulated vein using tip tracking. When satisfied with the position of the PVC, the participants would press an “evaluate position” button inside IVR and receive feedback on whether the PVC had been correctly placed before they moved on to the next scenario. A test conductor noted the time each participant spent in IVR and any technical problems that had arisen.

**Figure 2:**
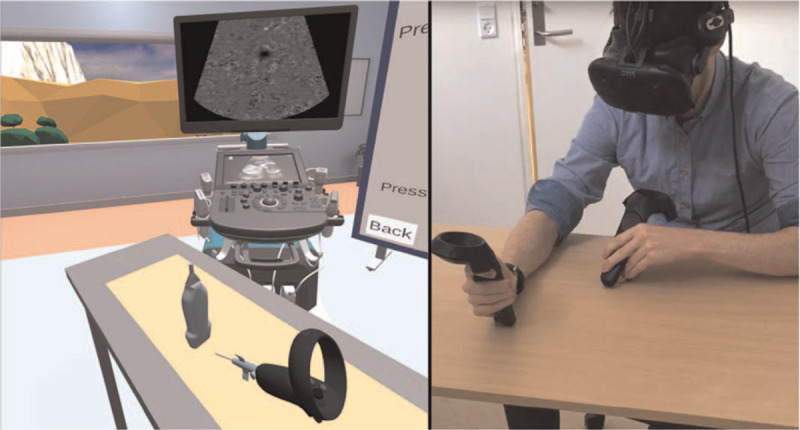
Setup of the IVR training for the intervention group. The left image shows the VR scenario that the participant in the right image is performing. IVR = immersive virtual reality, VR = virtual reality.

All 10 scenarios were similarly structured but they increased in difficulty, with the size of the vessel decreasing while its depth increased. The setup for the intervention is shown in Figure 2.

### 2.4 Assessment

**Figure 3:**
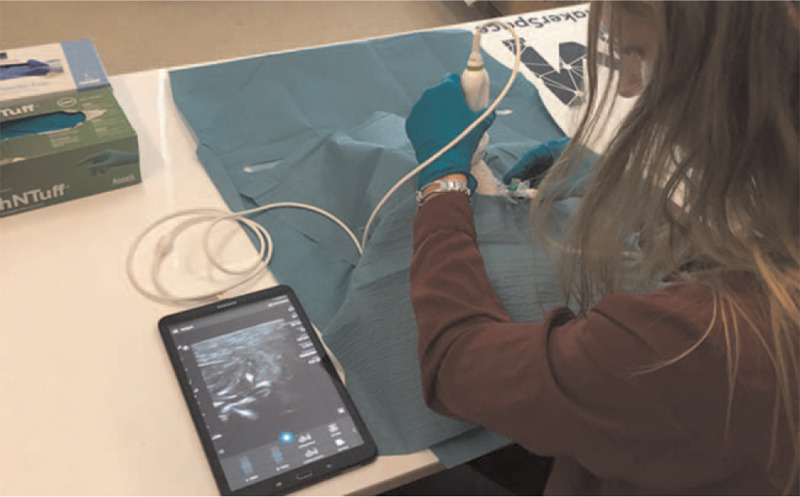
Setup of the assessment. A participant is performing US-guided PVC placement on the phantom. US = ultrasound, PVC = peripheral venous catheter.

All participants were subsequently assessed during a task in which they had to place up to 3 PVCs into a phantom using US guidance. Regardless of their assigned group, the participants had 15 minutes to cannulate the vessels in the phantom. To imitate veins, a modified version of Rippey et al phantom with chicken meat and water-filled balloons was used.^[26]^ The setup for the assessment is illustrated in Figure 3.

An attempt was deemed successful when the catheter tip was placed correctly and sufficiently inside the “vein” of the phantom. To increase the difficulty of the task, the sizes of the PVCs were increased. The 3 PVCs (B. Braun, Melsungen, Germany) used were 18G, 16G, and 14G. Scanning was performed using a small hand-held US scanner (Lumify, Philips, Amsterdam, Netherlands) with an Android-based Galaxy Tab A tablet (Samsung, Seoul, South Korea).

The participants were allowed to progress to the next PVC once they were satisfied with the placement of the current one. They were not allowed to go back to a PVC after they had deemed it successful. A blinded assessor evaluated the cannulation attempts when the participants had finished placing all 3 PVCs. To ensure a similar level of difficulty for each participant, the balloons that formed the phantom's “veins” were changed and the new balloons were placed in pre-made indentations. This gave the assessor the opportunity to assess all cannulation attempts and collect the following data: scanning time before each PVC insertion, number of surface punctures in the phantom, vessel punctures in the phantom, time used for US tip tracking, and number of successful cannulations. The numbers of successful cannulations and surface punctures were converted to proportions, the former describing the number of assessor-approved cannulations and the latter, the number of surface punctures correlated with successful cannulations.

Participants received no guidance or feedback on the placement until after the assessment so that they would not know during the task whether they had placed the PVCs correctly.

### 2.5 Power calculation

The power calculation for this study showed that 8 participants per group were needed to show a significant difference in the results of the study. An unpublished master thesis on the effect of IVR for basic US skill acquisition for medical studentsfound a 10% difference in the final score between the study groups (E.Q. Kristensen, MD, unpublished data, January 2019). This difference, a standard deviation of 7%, and a power of 80% were applied to a standard power calculation to estimate the sample size.

Data were analyzed using Stata IC version 16.0 (StataCorp LLC, Texas, USA). Results were collected as numerical or binary (yes/no) data. Means and standard deviations were calculated as descriptive parameters.

Quantile-quantile (QQ)-plots and Shapiro–Wilk test were used to test for normal distribution. Fisher exact test was used to test statistical significance for successful cannulations and surface punctures of the phantom. The Wilson score interval was used to calculate 95% confidence intervals for both types of proportions. Two-sample *t* tests were used for procedure time. The number of successful cannulations was summarized as frequencies, and absolute proportions between the 2 groups were calculated and compared. A *P* value <.05 was considered statistically significant.

The study was conducted in accordance with the Helsinki Declaration. No ethical approval was needed due to the study's educational focus. Informed consent was obtained from all participants prior to data collection.

## 3 Results

The data were collected at SDU in Odense, Denmark on March 5, 2019. All 19 participants completed the course, with 10 participants in the IVR group and 9 in the control group.

All of the participants’ data were used, except for 1 data point regarding the 3rd PVC of a participant from the control group. This data point was excluded because the participant failed to perform this attempt within the predefined 15-minute time frame. Hence, only 8 out of 9 observations were used for the 3rd PVC in the control group.

The proportion of successful peripheral venous cannulations for the IVR group was 22 out of 30 (73%). For the control group, 6 out of 27 (22%) cannulations were successful. Thus, an absolute difference of 51% in the total amount of successful cannulations was found between the 2 groups. The proportion of successful cannulations was significantly higher for the IVR group (*P* ≤ .001).

For the 1st, 2nd, and 3rd PVC, the proportions of successful peripheral venous cannulations in the IVR group were (100%), 8 out of 10 (80%), and 4 out of 10 (40%), respectively, compared to 4 out of 9 (44%), 2 out of 9 (22%), and 0 out of 9 (0%) for the control group. The percentage-wise absolute differences in successful cannulations between the groups were 56% for the 1st PVC, 58% for the 2nd PVC, and 40% for the 3rd PVC. Furthermore, the proportion of successful cannulations was significantly higher in the IVR group compared to the control group for the 1st and 2nd PVC (*P* = .011, *P* = .023), but not for the 3rd PVC (*P* = .087). The results are shown in Table 2.

**Table 2:** Results of Fisher exact tests for the proportion of successful cannulations and surface punctures.

Parameters	Results for intervention group	Results for control group	Absolute difference	*P* value
	Successful cannulations			
Cannula 1 [95% CI]	10/10 (100%) [0.72; 1]	4/9 (44.4%) [0.19; 0.73]	55.6%	.011
Cannula 2 [95% CI]	8/10 (80%) [0.49; 0.94]	2/9 (22.2%) [0.06; 0.55]	57.8%	.023
Cannula 3 [95% CI]	4/10 (40%) [0.17; 0.69]	0/9 (0%) [0.0; 0.29]	40%	.087
Total [95% CI]	22/30 (73.3%) [0.56; 0.86]	6/27 (22.2%) [0.11; 0.41]	51.1%	<.001
	Surface punctures			
Cannula 1 [95% CI]	10/12 (83.3%) [0.55; 0.95]	4/23 (17.4%) [0.07; 0.37]	65.9%	<.001
Cannula 2 [95% CI]	8/13 (61.5%) [0.36; 0.82]	2/23 (8.7%) [0.02; 0.27]	52.8%	.001
Cannula 3 [95% CI]	4/14 (28.6%) [0.12; 0.55]	0/10 (0%) [0.0; 0.28]	28.6%	.114
Total [95% CI]	22/39 (56.4%) [0.41; 0.71]	6/56 (10.7%) [0.05; 0.22]	45.7%	<.001

For “Successful cannulations,” the numerator is the absolute number of successful cannulations and the denominator is the highest possible number. For “Surface punctures,” the numerator indicates the number of surface punctures compared to successful cannulations and the denominator is the total number. Proportions are given in round brackets. 95% confidence intervals from 0 to 1 are given in square brackets. The 1st and 2nd peripheral venous catheter and total are significant for both the proportion of successful cannulations and surface punctures. CI = confidence interval.

The proportion of surface punctures compared to successful cannulations for the IVR group was 10 out of 12 (83%), 8 out of 13 (62%), and 4 out of 14 (29%) for the 1st, 2nd, and 3rd PVC, respectively, and 22 out of 39 (56%) in total. In other words, 83% of the surface punctures for the 1st PVC in the IVR group were correlated with a successful cannulation. For this study, surface punctures were defined as the instance when the needle tip of the PVC penetrated the surface of the phantom, but not the vein inside the phantom.

For the control group, the proportions were 4 out of 23 (17%), 2 out of 23 (9%), and 0 out of 10 (0%) for the 1st, 2nd, and 3rd PVC, respectively, and 6 out of 56 (11%) in total. The percentage-wise absolute differences in surface punctures between the groups were 66%, 53%, and 29% for the 1st, 2nd, and 3rd PVC, respectively, while 46% in the total amount of surface punctures.

Significant differences between the groups were found for the 1st and 2nd PVC (*P* ≤ .001 and *P* ≤ .001, respectively), but not for the 3rd (*P* = .114). Overall, the proportion of surface punctures was significantly lower in the IVR group compared to the control group (*P* ≤ .001). The results are shown in Table 2.

**Figure 4:**
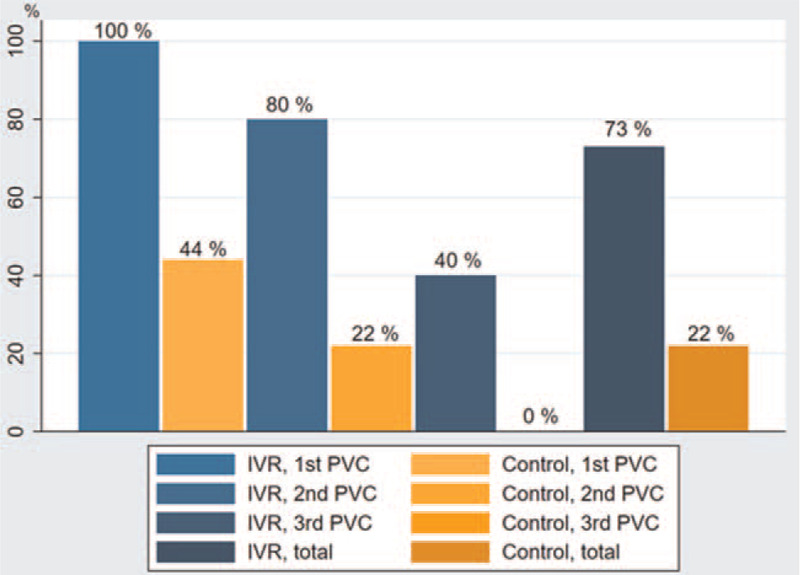
Bar diagram on procedure times. 95% confidence intervals are stated in square brackets. No significant difference was found between the groups. PVC = peripheral venous catheter, IVR = immersive virtual reality.

Data for procedure times were considered normally distributed as points from the 12 different QQ-plots formed roughly straight lines and Shapiro–Wilk test was non-significant (*P* > .05). The only exception was data from “Needle 1, prescan VR” (*P* ≤ .001) (see figures A-L, Supplemental Digital Content 1 for illustration of the QQ-plots). Mean prescan time for the IVR group was 38.6 (95% CI [14.4–62.8]), 37.0 (95% CI [15.8–58.2]), and 46.5 (95% CI [16.8–76.3]) seconds for the 1st, 2nd, and 3rd PVC, respectively. For the control group, mean prescan time for the same PVCs was 48.3 (95% CI [39.2–57.4)], 26.6 (95% CI [17.8–35.4]), and 42.3 (95% CI [27.8–56.7]) seconds, respectively. Mean tip tracking time for the IVR group was 142.3 (95% CI [85.0–199.6]), 206.0 (95% CI [120.8–291.2]), and 208.2 (95% CI [85.6–330.8]) seconds for the 1st, 2nd, and 3rd PVC, respectively. For the control group, the mean tip tracking time for the same PVCs was 195.4 (95% CI [119.4–271.5]), 267.4 (95% CI [125.7–409.2]), and 177.3 (95% CI [81.5–273.0]) seconds, respectively. No significant differences in prescan and tip tracking times were observed between the 2 groups (*P* > .05) (see table, Supplemental Digital Content 2 for the results of procedure time results). A bar graph including means for the prescan and tip tracking times across all PVCs and between both groups, measured in seconds, is illustrated in Figure 4.

## 4 Discussion

This pilot study showed that IVR learning had a positive and significant effect on US-guided PVC placements on a phantom following the training. The IVR learning also had a significant impact on the proportion of unnecessary surface punctures. No significant difference in procedure time between the groups was observed. To the best of our knowledge, this is the first study to explore the effect of IVR on US-guided PVC placement.

Skill acquisition through IVR has previously been demonstrated in a randomized controlled trial^[20]^: This study found that 40 surgical trainees’ ability to tie a single-handed reef knot improved significantly after a 15-minute IVR lesson compared to after watching a video demonstration and again after subsequent face-to-face teaching with a blinded instructor. These results^[20]^ indicate that IVR training has a significantly higher impact on learning efficacy than watching a video demonstration, and that not even subsequent training with an instructor compensates for the difference in ability. Although the article does not entirely fall under the same field as this study, it is currently 1 of the most comparable of the use of IVR in education.

It is important to acknowledge other educational tools in teaching the US: A study on US-guided PVC placement comparing face-to-face teaching, e-learning, and no specific education found that face-to-face teaching resulted in the best success rate scores and had the highest rating amongst the included medical students (n = 30).^[27]^ Furthermore, improvement in PVC placement was significantly higher after face-to-face teaching compared to no education and trended towards outperforming e-learning. These results suggest that blended learning could be the preferred approach in a course on US-guided PVC placement.

VR-simulated procedures have high repeatability, which makes it easy for novices to practice a procedure several times and learn from their mistakes in a safe environment. A large range of IVR equipment is commercially available in the price range of 180–900 EUR. The cost of the HTC Vive head-mounted display used in this study, including the controllers, was 500 EUR. The future cost of software and license is estimated at 1000 EUR per year for general use. Compared to other US simulation equipment, this setup was relatively inexpensive, making it feasible for a university to purchase materials for a larger setup that enables more students to practice simultaneously, possibly lowering expenses on instructors and equipment.

The internal validity of this study was increased using the same IVR simulation and type of intervention phantom to assess each participant. Resetting the setup between each participant by changing the balloons that formed the vessels in the phantom ensured that every participant had the same experience and that the data collected were comparable. Using the same blinded assessor for each evaluation minimized the risk of observation bias.

An important limitation was the lack of further training in the control group. A third group training on the phantom itself would have increased the strength of our study and allowed us to compare the effect of IVR training with that of another form of training.

Recruiting students through the university's internal communication platform might have entailed a risk of selection bias. Students with interest in research or VR were more likely to volunteer as participants, which might have led to a higher proportion of successful cannulations overall and therefore decreased the study's external validity. However, any difference in performance should be negligible since the proportion of successful cannulations is expected to be higher, regardless of the group. The risk of selection bias was further minimized by blinding the participants.

Although the use of a standardized intervention phantom can be beneficial in training novices and performing IVR simulations, it is not comparable to a real patient in a clinical setting. Future research within this subject should focus on optimizing the method so that the entire process of PVC placement is covered and includes identifying an appropriate anatomical site, creating stasis, palpating a vein, disinfecting the area, etc. Another aspect to investigate is the clinical potential of using IVR in a US-guided PVC placement course. A randomized controlled study could clarify the potential impact on patient care and economy when healthcare professionals attend a course in US-guided PVC placement using either IVR or conventional methods. Including these factors would increase the clinical relevance, and the method could then be used to teach PVC placement to the relevant personnel such as ward nurses who are inexperienced in the US.

In future research, a larger sample size from a broader population (profession-wise) would increase the validity of the results. Adding a third group with instructor-led training and a more clinically relevant outcome, such as successful PVC placements in actual patients would also increase the strength of a future study.

## 5 Conclusion

This pilot study showed that adding an IVR-based training simulation that focused on tip tracking skills to an existing e-learning curriculum significantly increased the learning efficacy for US-guided PVC placement for medical students: 73% of all cannulations were successful in the intervention group, compared to 22% in the control group. The proportion of surface punctures was also significantly lower for the IVR group, but no significant difference for procedure time was found between the groups.

As this was a pilot study with a small sample size of medical students, further research is needed to clarify the clinical implications of using IVR as a supplementary training tool when teaching US-guided PVC placement. Further research should include a larger and more generalizable study population and a more clinically relevant outcome such as successful US-guided PVC placements in patients.

## Acknowledgments

The authors would like to thank BSc. Medialogy Boas K. Christiansen, Emil F. Skov, and Mikkel Laursen from Aalborg University for participating in execution and data collection. All technical equipment used for IVR training, including the IVR simulation, were borrowed from VitaSim and SDU. Classroom facilities were provided by SDU.

## Author contributions

**Conceptualization:** Rune Overgaard Jensen, Rasmus Jørgensen, Ole Graumann.

**Data curation:** Nanna Lind Andersen.

**Formal analysis:** Nanna Lind Andersen.

**Investigation:** Rune Overgaard Jensen, Rasmus Jørgensen.

**Methodology:** Rune Overgaard Jensen, Stefan Posth, Christian B. Laursen, Rasmus Jørgensen, Ole Graumann.

**Project administration:** Rune Overgaard Jensen, Ole Graumann.

**Resources:** Rune Overgaard Jensen, Rasmus Jørgensen.

**Software:** Rune Overgaard Jensen.

**Supervision:** Rune Overgaard Jensen, Stefan Posth, Christian B. Laursen, Ole Graumann.

**Validation:** Rune Overgaard Jensen, Ole Graumann.

**Visualization:** Nanna Lind Andersen, Stefan Posth, Christian B. Laursen.

**Writing – original draft:** Nanna Lind Andersen.

**Writing – review & editing:** Nanna Lind Andersen, Rune Overgaard Jensen, Stefan Posth, Christian B. Laursen, Ole Graumann.

## Supplementary Material

Supplemental Digital Content

## Supplementary Material

Supplemental Digital Content

## References

[1] Scholten HJ Pourtaherian A Mihajlovic N Korsten HHM Bouwman RA. Improving needle tip identification during ultrasound-guided procedures in anaesthetic practice. Anaesthesia 2017;72:889–904. doi:10.1111/anae.13921. 28542716 10.1111/anae.13921

[2] Duran-Gehring P Bryant L Reynolds JA Aldridge P Kalynych CJ Guirgis FW. Ultrasound-guided peripheral intravenous catheter training results in physician-level success for emergency department technicians. J Ultrasound Med 2016;35:2343–52. doi:10.7863/ultra.15.11059. 27629755 10.7863/ultra.15.11059

[3] Alexandrou E Ray-Barruel G Carr PJ. Use of short peripheral intravenous catheters: characteristics, management, and outcomes worldwide. J Hosp Med 2018;13: doi:10.12788/jhm.3039. 10.12788/jhm.3039 29813140

[4] Fields JM Piela NE Au AK Ku BS. Risk factors associated with difficult venous access in adult ED patients. Am J Emerg Med 2014;32:1179–82. doi:10.1016/j.ajem.2014.07.008. 25171796 10.1016/j.ajem.2014.07.008

[5] Cooke M Ullman AJ Ray-Barruel G Wallis M Corley AJ Rickard CM. Not "just” an intravenous line: consumer perspectives on peripheral intravenous cannulation (PIVC). An international cross-sectional survey of 25 countries. PLoS ONE 2018;13:e0193436. 29489908 10.1371/journal.pone.0193436 PMC5831386

[6] Hadaway L. Short peripheral intravenous catheters and infections. J Infus Nurs 2012;35:230–40. doi:10.1097/NAN.0b013e31825af099. 22759827 10.1097/NAN.0b013e31825af099

[7] Neuhaus D. Intraosseous infusion in elective and emergency pediatric anesthesia: when should we use it? Curr Opin Anaesthesiol 2014;27:282–7. doi:10.1097/ACO.0000000000000069. 24651308 10.1097/ACO.0000000000000069

[8] Voigts B Abolmaali N Stelzner C Schellong SM. Imaging representation of peripheral veins. Internist (Berl) 2017;58:796–804. doi:10.1007/s00108-017-0281-5. 28656317 10.1007/s00108-017-0281-5

[9] Chang P-H Chen Y-J Chang K-V. Ultrasound measurements of superficial and deep masticatory muscles in various postures: reliability and influencers. Sci Rep 2020;10:14357 doi:10.1038/s41598-020-71378-z. 32873849 10.1038/s41598-020-71378-z PMC7463001

[10] Chiu YH Chang KV Chen IJ Wu WT Özçakar L. Utility of sonoelastography for the evaluation of rotator cuff tendon and pertinent disorders: a systematic review and meta-analysis. Eur Radiol 2020;30:6663–72. doi:10.1007/s00330-020-07059-2. 32666319 10.1007/s00330-020-07059-2

[11] van Loon FHJ Buise MP Claassen JJF Dierick-van Daele ATM Bouwman ARA. Comparison of ultrasound guidance with palpation and direct visualisation for peripheral vein cannulation in adult patients: a systematic review and meta-analysis. Br J Anaesth 2018;121:358–66. doi:10.1016/j.bja.2018.04.047. 30032874 10.1016/j.bja.2018.04.047

[12] McCarthy ML Shokoohi H Boniface KS. Ultrasonography versus landmark for peripheral intravenous cannulation: a randomized controlled trial. Ann Emerg Med 2016;68:10–8. doi:10.1016/j.annemergmed.2015.09.009. 26475248 10.1016/j.annemergmed.2015.09.009

[13] Vitto MJ Myers M Vitto CM Evans DP. Perceived difficulty and success rate of standard versus ultrasound-guided peripheral intravenous cannulation in a Novice Study Group: a randomized crossover trial. J Ultrasound Med 2016;35:895–8. doi:10.7863/ultra.15.06057. 27009314 10.7863/ultra.15.06057

[14] Kanipe W Shobe K Li Y Kime M Smith-Miller CA. Evaluating the efficacy and use of vein visualization equipment among clinical nurses in an intermediate care environment. J Infus Nurs 2018;41:253–8. doi:10.1097/NAN.0000000000000286. 29958262 10.1097/NAN.0000000000000286

[15] Jeon Y Choi S Kim H. Evaluation of a simplified augmented reality device for ultrasound-guided vascular access in a vascular phantom. J Clin Anesth 2014;26:485–9. doi:10.1016/j.jclinane.2014.02.010. 25204510 10.1016/j.jclinane.2014.02.010

[16] Breslin R Collins K Cupitt J. The use of ultrasound as an adjunct to peripheral venous cannulation by junior doctors in clinical practice. Med Teach 2018;40:1275–80. doi:10.1080/0142159X.2018.1428737. 29385869 10.1080/0142159X.2018.1428737

[17] Loon FHV Scholten HJ Erp IV Bouwman AR Daele ATDV. Establishing the required components for training in ultrasoundguided peripheral intravenous cannulation: a systematic review of available evidence. Med Ultrason 2019;21:464–73. doi:10.11152/mu-2120. 31765456 10.11152/mu-2120

[18] Frederiksen JG Sørensen SMD Konge L. Cognitive load and performance in immersive virtual reality versus conventional virtual reality simulation training of laparoscopic surgery: a randomized trial. Surg Endosc 2020;34:1244–52. doi:10.1007/s00464-019-06887-8. 31172325 10.1007/s00464-019-06887-8

[19] Maresky HS Oikonomou A Ali I Ditkofsky N Pakkal M Ballyk B. Virtual reality and cardiac anatomy: exploring immersive three-dimensional cardiac imaging, a pilot study in undergraduate medical anatomy education. Clin Anat 2019;32:238–43. doi:10.1002/ca.23292. 30295333 10.1002/ca.23292

[20] Yoganathan S Finch DA Parkin E Pollard J. 360° virtual reality video for the acquisition of knot tying skills: a randomised controlled trial. Int J Surg 2018;54:24–7. doi:10.1016/j.ijsu.2018.04.002. 29649669 10.1016/j.ijsu.2018.04.002

[21] Latham K Kot P Wariach A Al-Jumeily D Puthuran M Chandran A. A Review on the Development of a Virtual Reality Learning Environment for Medical Simulation and Training. Rome, Italy: VISUAL; 2019.

[22] Adhikari S Schmier C Marx J. Focused simulation training: emergency department nurses’ confidence and comfort level in performing ultrasound-guided vascular access. J Vasc Access 2015;16:515–20. doi:10.5301/jva.5000436. 26109540 10.5301/jva.5000436

[23] İsmailoğlu EG Orkun N Eşer İ Zaybak A. Comparison of the effectiveness of the virtual simulator and video-assisted teaching on intravenous catheter insertion skills and self-confidence: a quasi-experimental study. Nurse Educ Today 2020;95:104596 doi:10.1016/j.nedt.2020.104596. 33002745 10.1016/j.nedt.2020.104596

[24] Todsen T Foss KT Bessmann EL. Acute Ultrasound [Akut Ultralyd]. CEKU Productions. Accessed May 1, 2020. http://akutul.cekuapp.dk/#intro.

[25] Weile J Todsen T. Vascular Access Instructional Video [Vaskulær Adgang Instruktionsvideo]. CEKU Productions. Accessed May 1, 2020. http://akutul.cekuapp.dk/#vasc_video.

[26] Rippey JCR Blanco P Carr PJ. An affordable and easily constructed model for training in ultrasound-guided vascular access. J Vasc Access 2015;16:422–7. doi:10.5301/jva.5000384. 26349885 10.5301/jva.5000384

[27] Lian A Rippey JCR Carr PJ. Teaching medical students ultrasound-guided vascular access – which learning method is best? J Vasc Access 2017;18:255–8. doi:10.5301/jva.5000730. 28430318 10.5301/jva.5000730

