# Supplementary figures and images for: Teaching ultrasound-guided peripheral venous catheter placement through immersive virtual reality: An explorative pilot study
Source: Medicine (Baltimore). 2021 Jul 9;100(27):e26394. doi: 10.1097/MD.0000000000026394 (PMC8270624; doi:10.1097/MD.0000000000026394)

**Supplemental Digital Content 1: QQ-plots for prescan and tip tracking time**


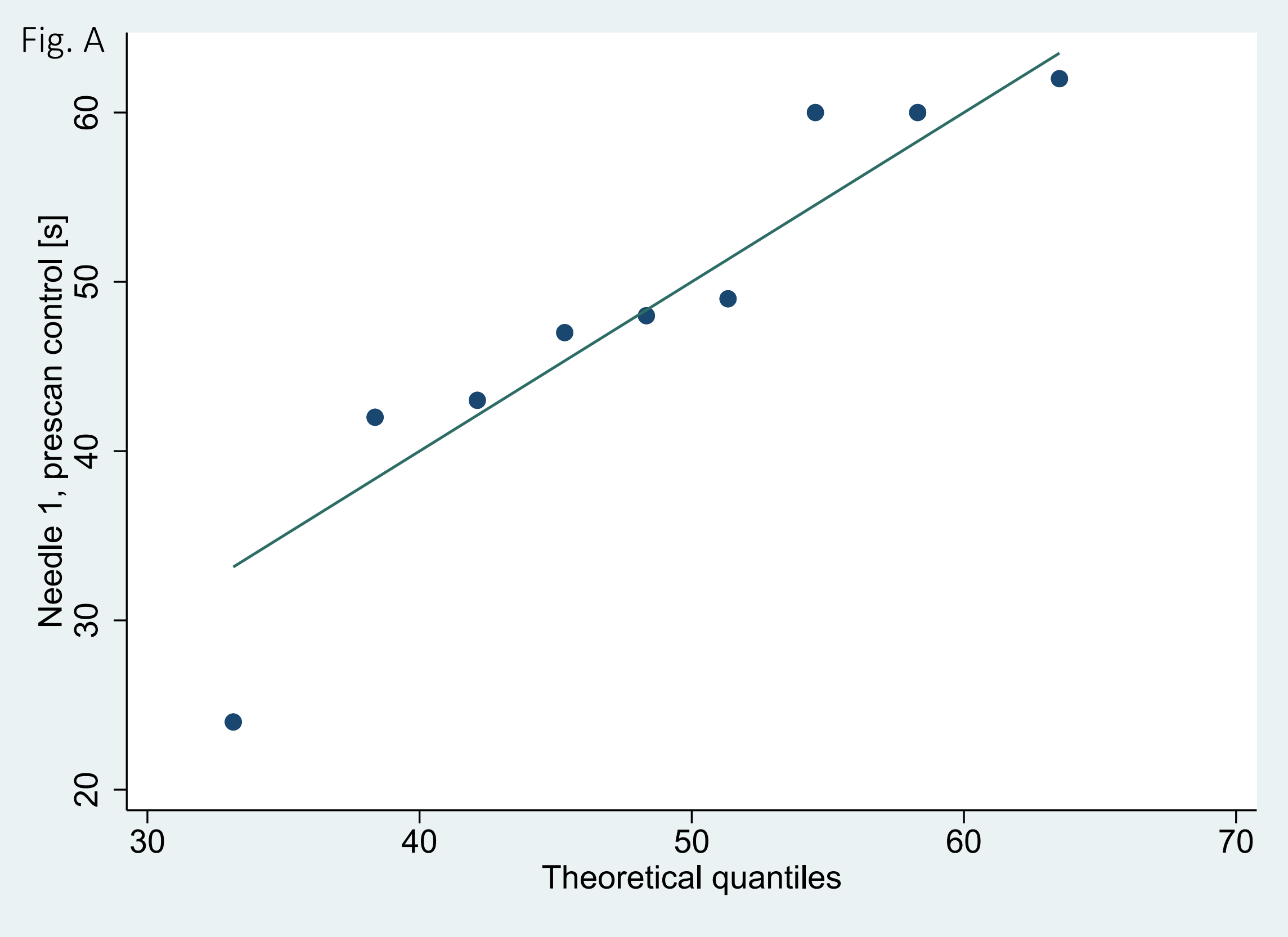

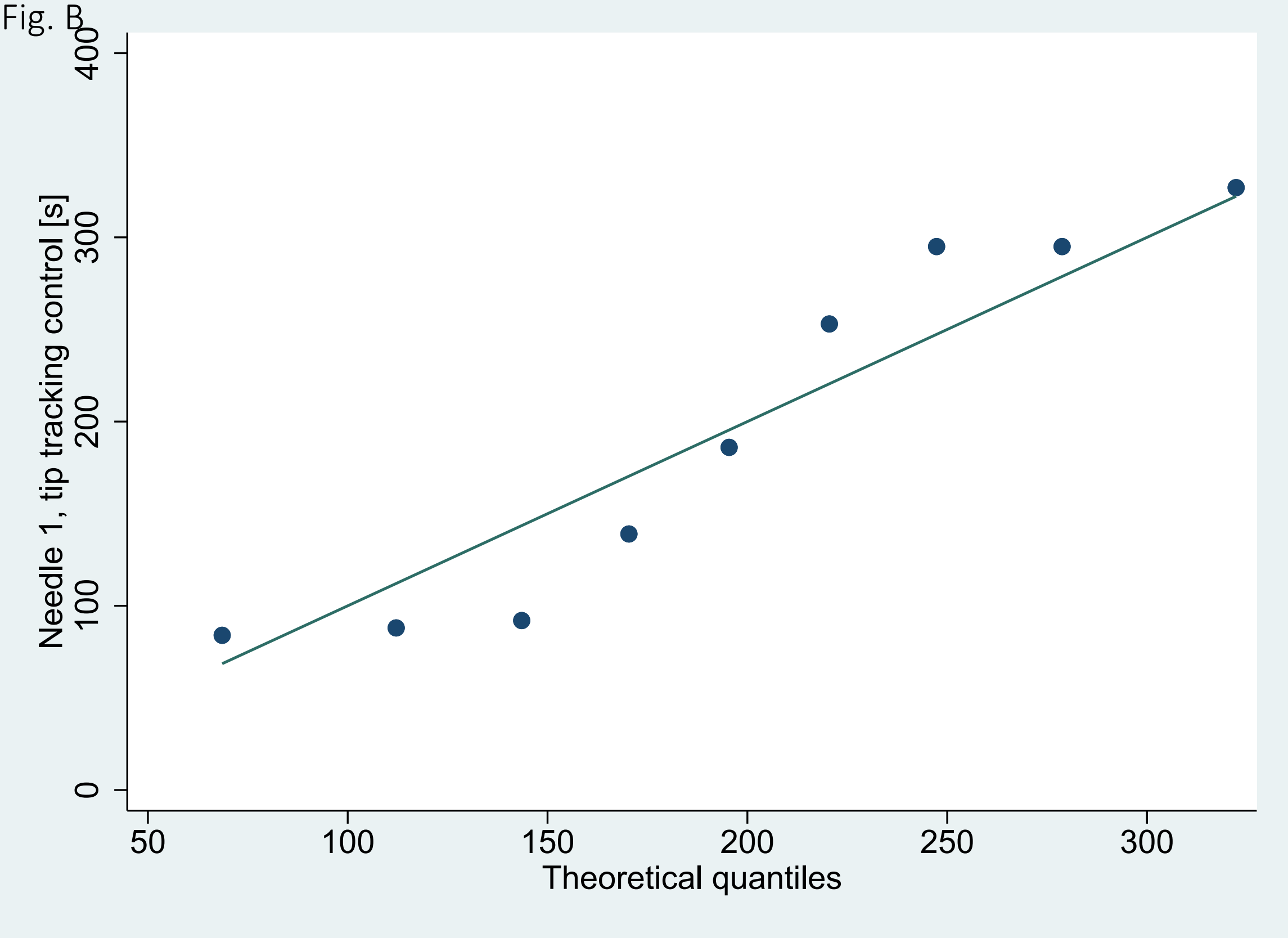


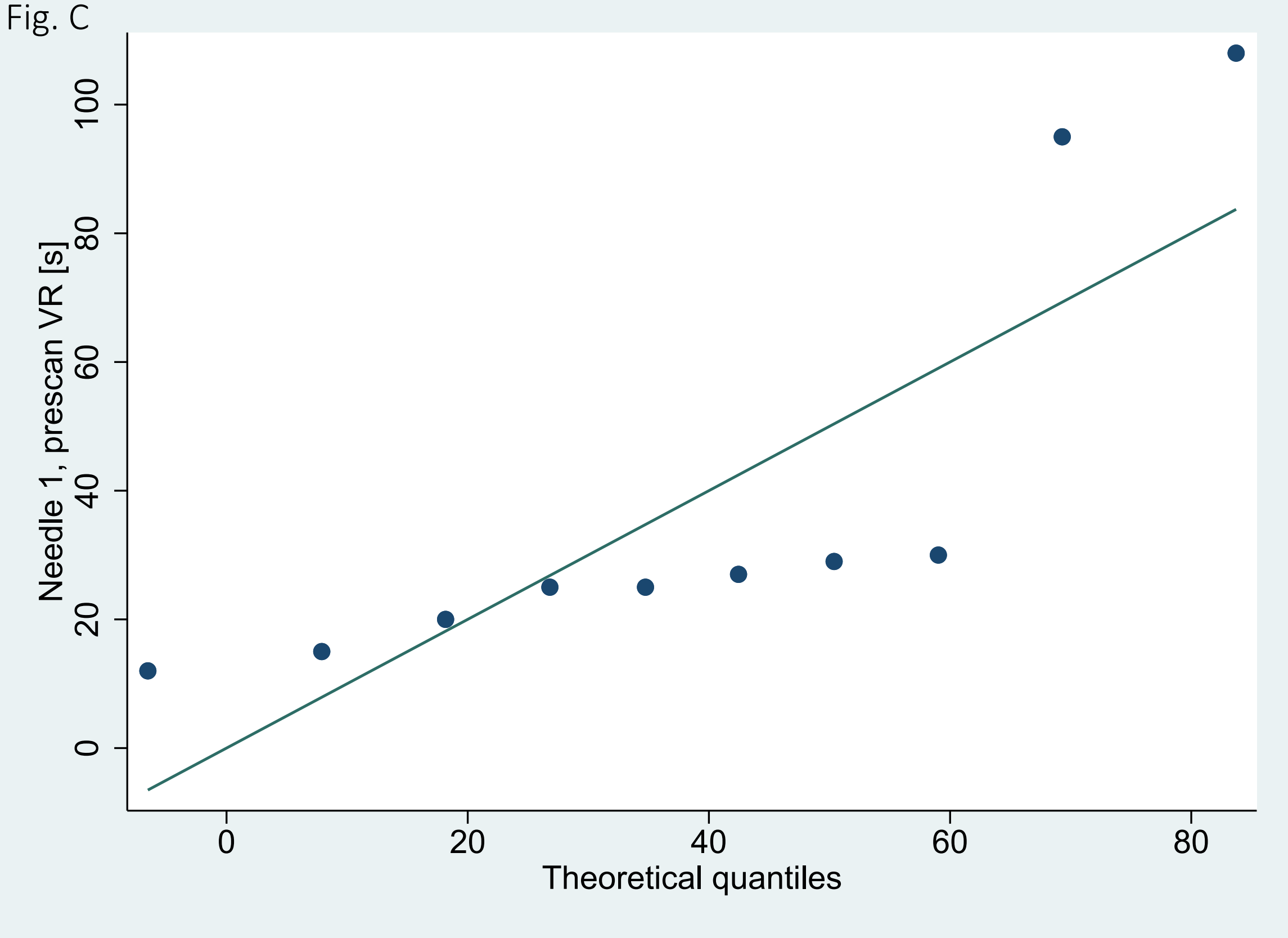

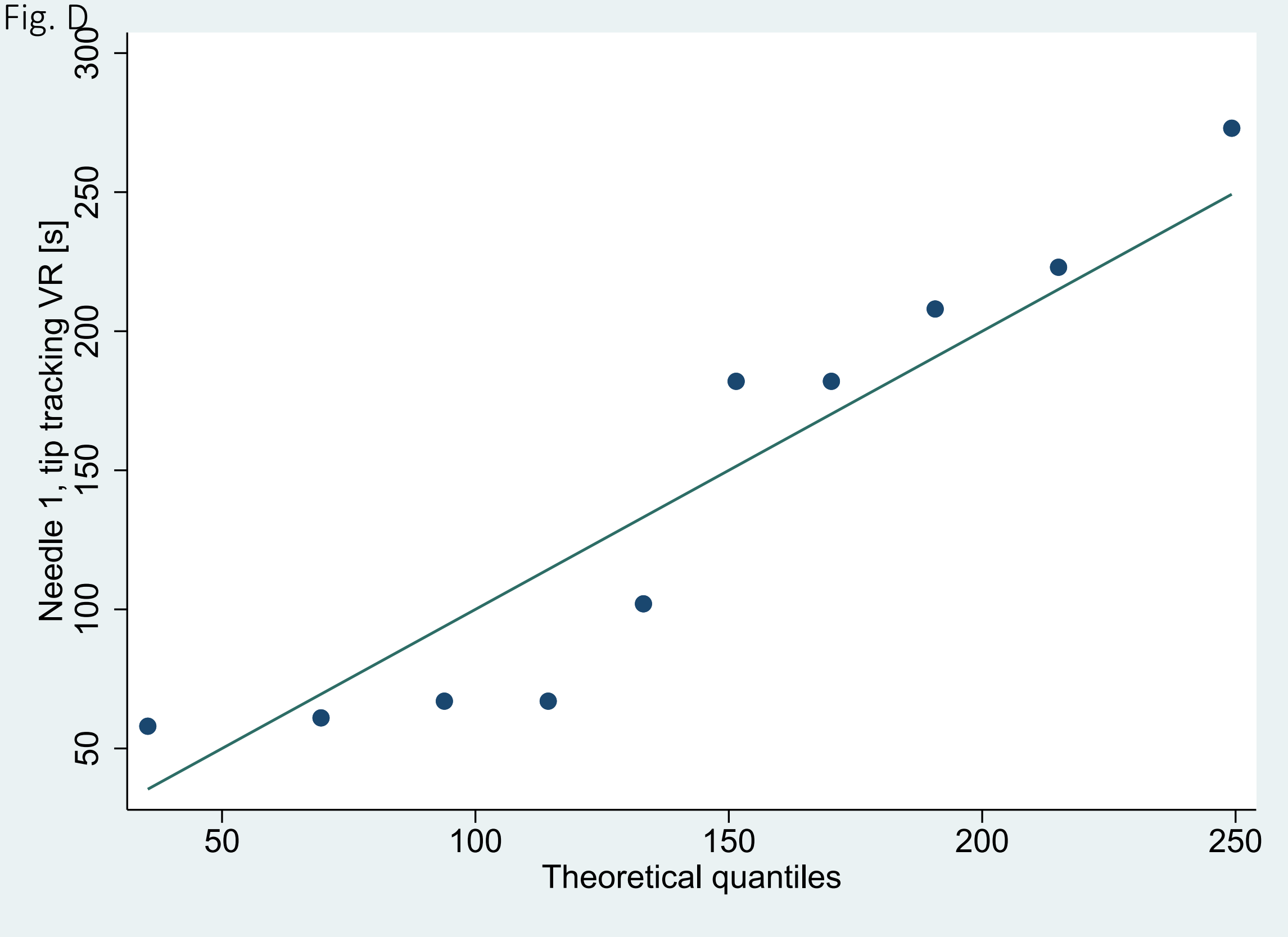


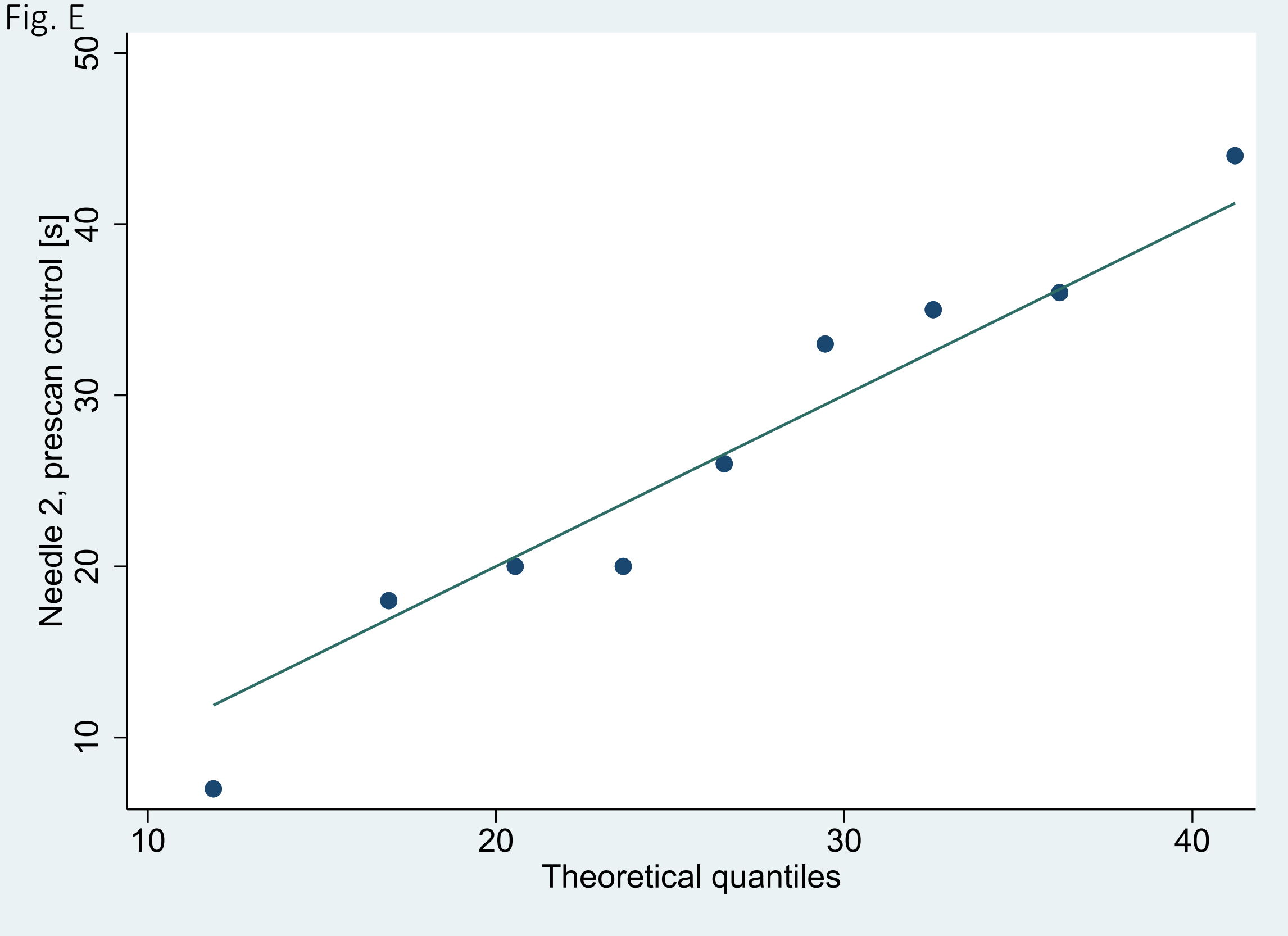

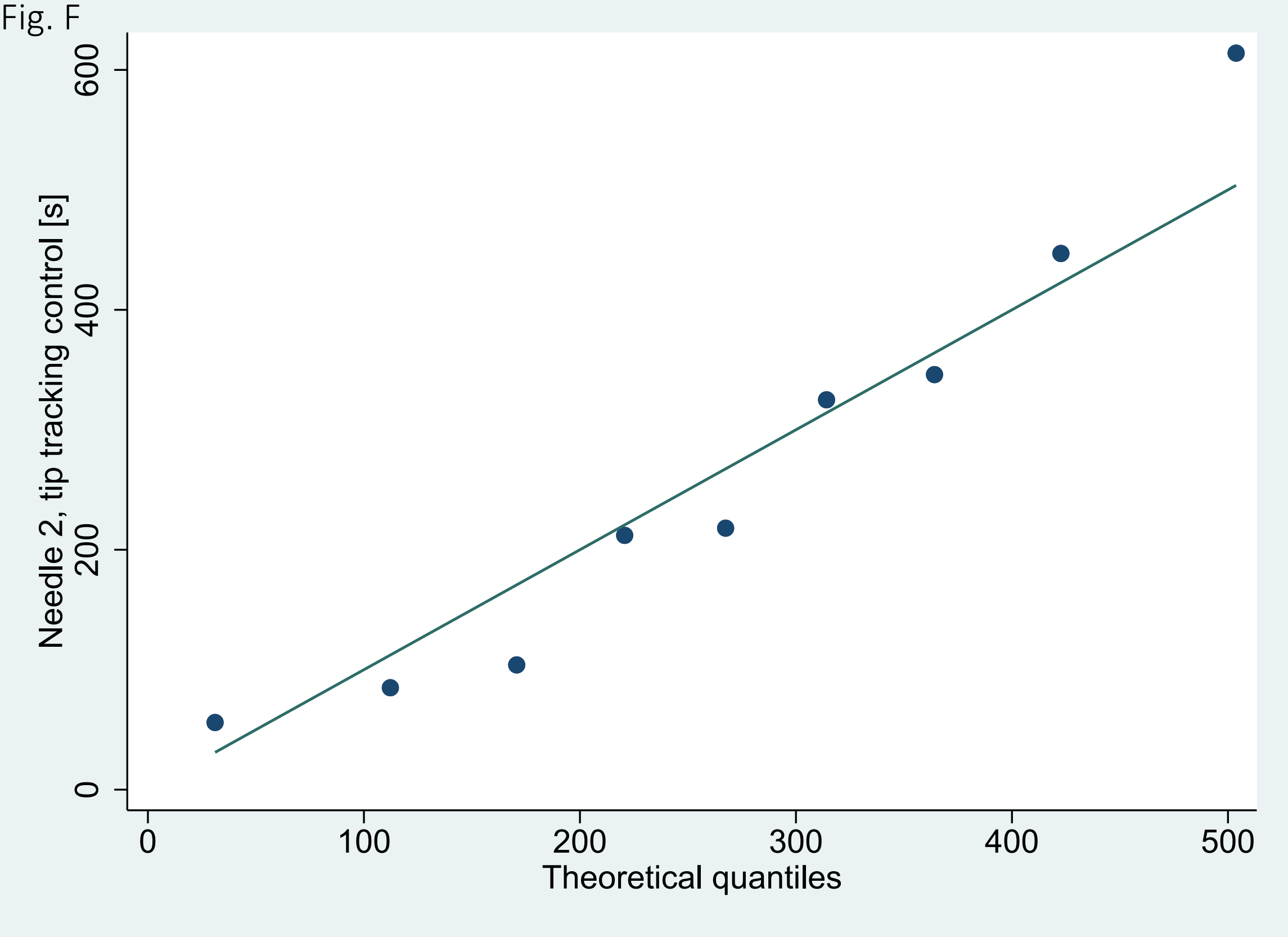


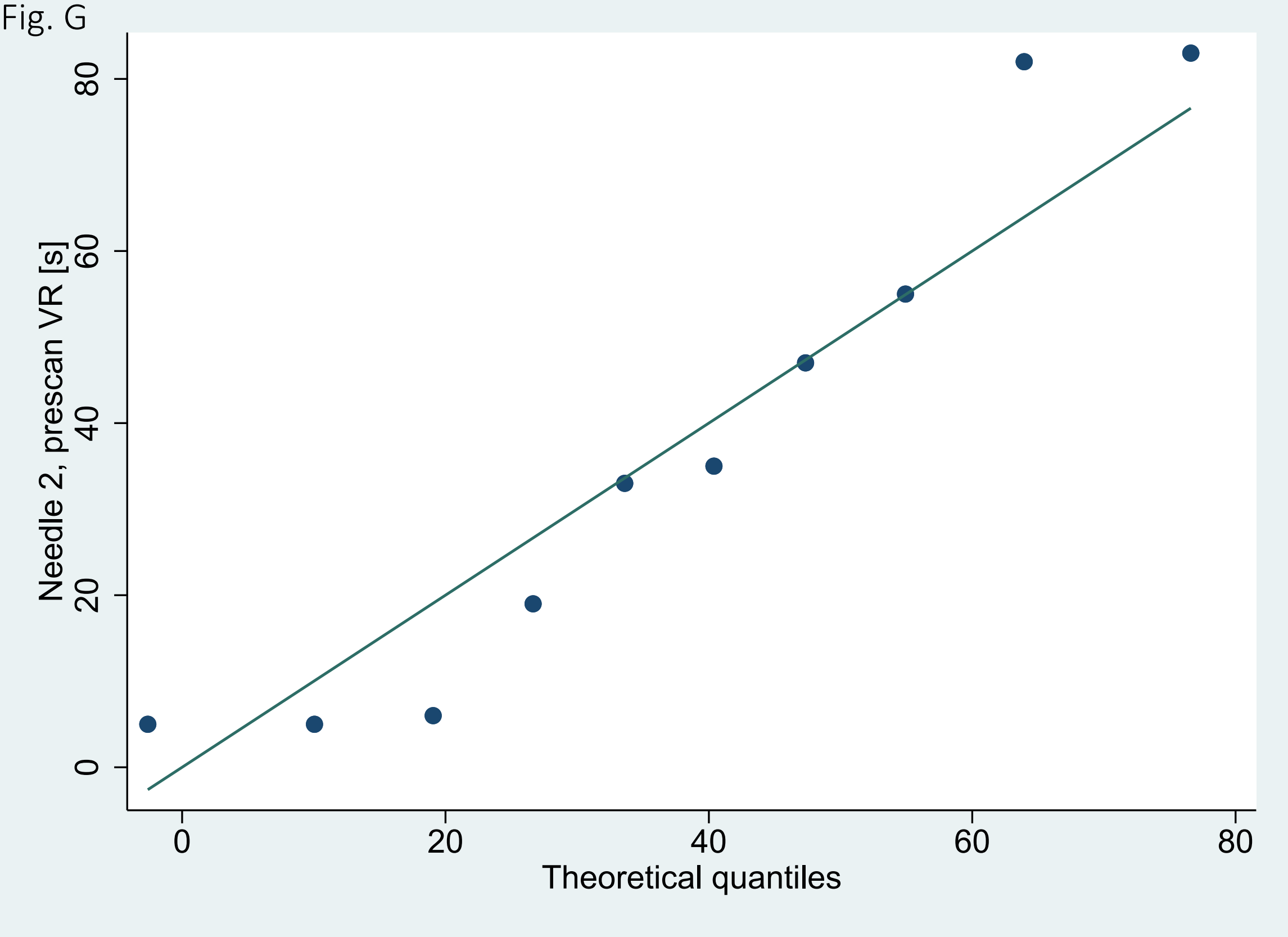

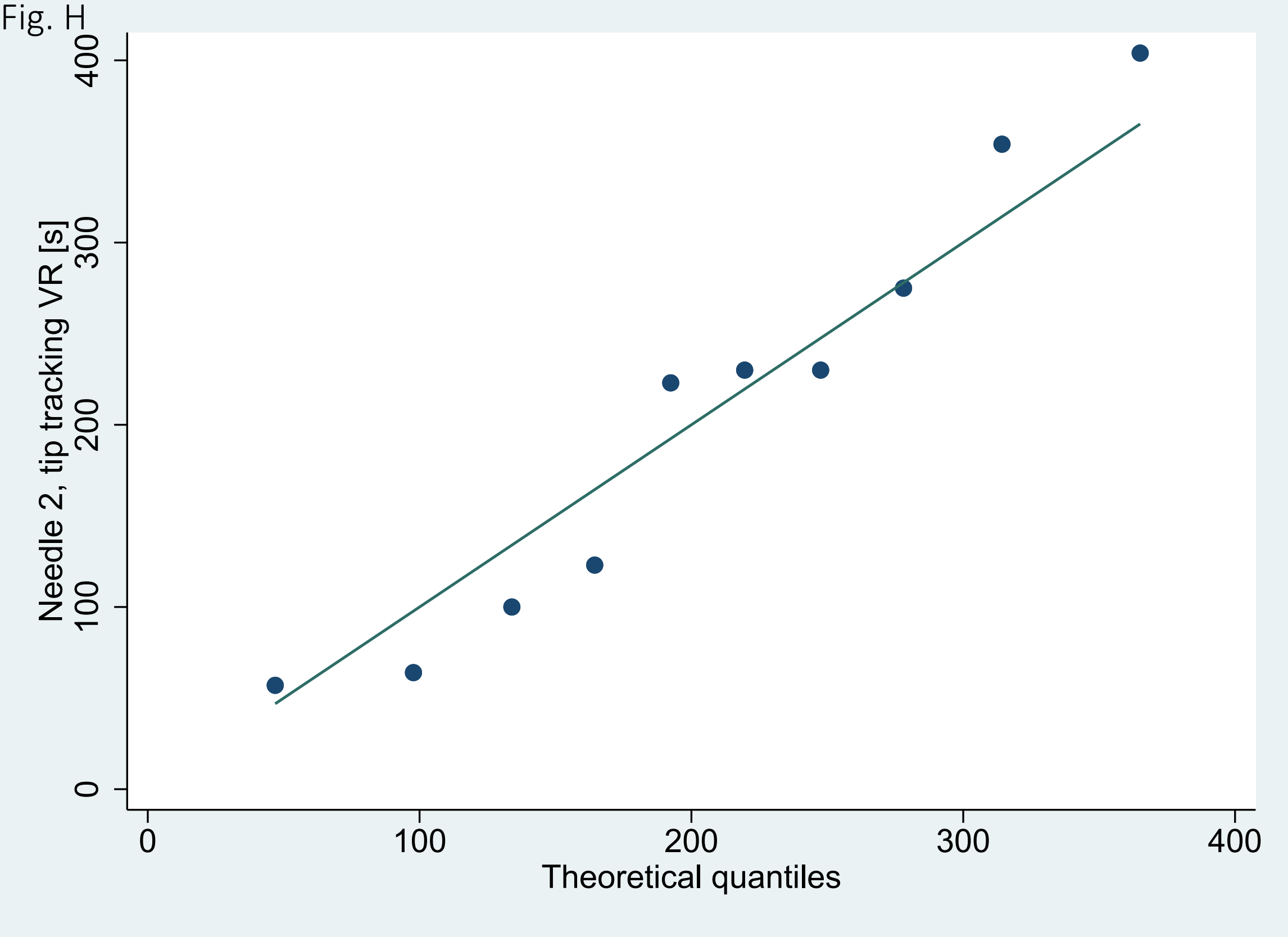


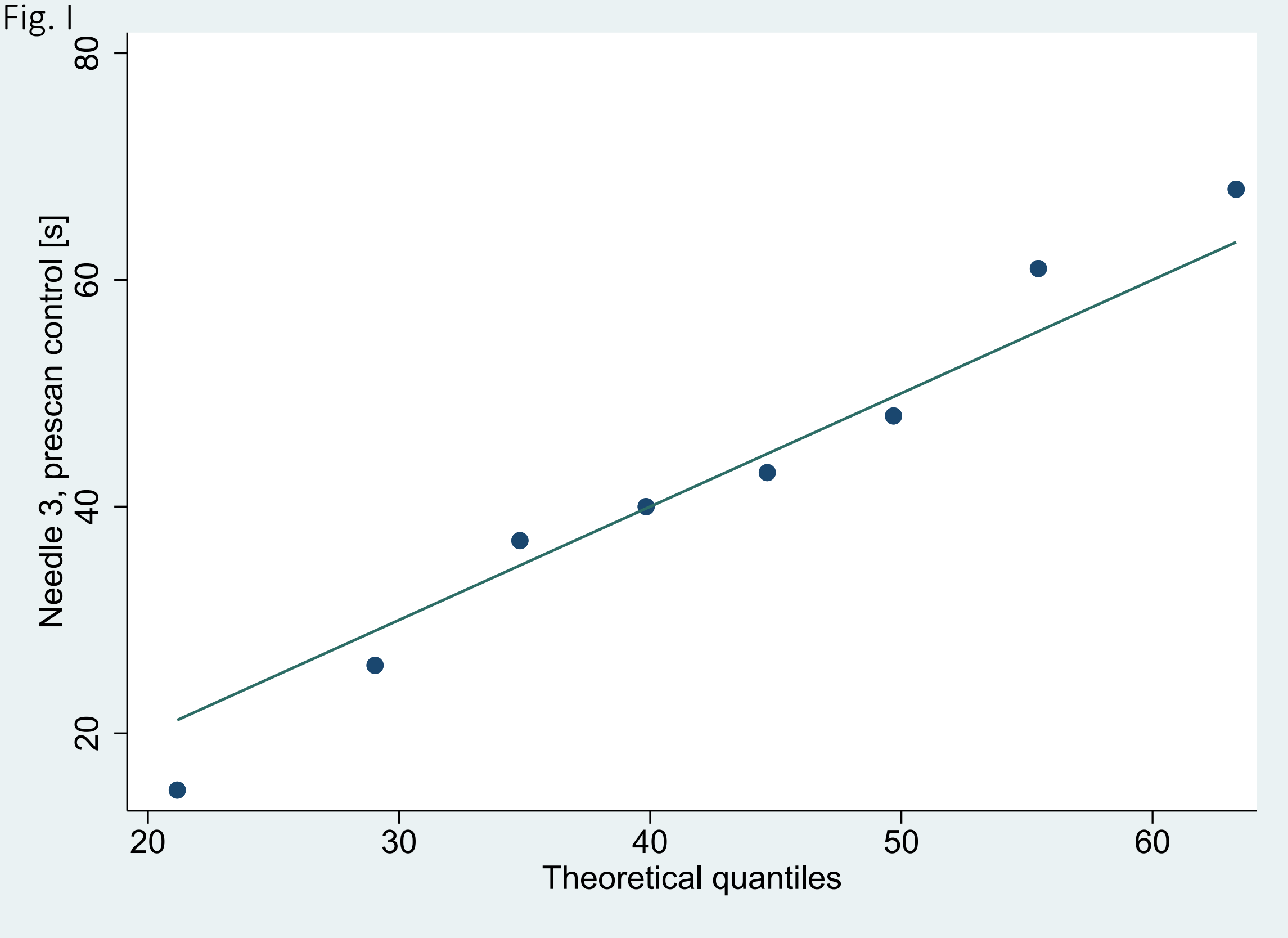

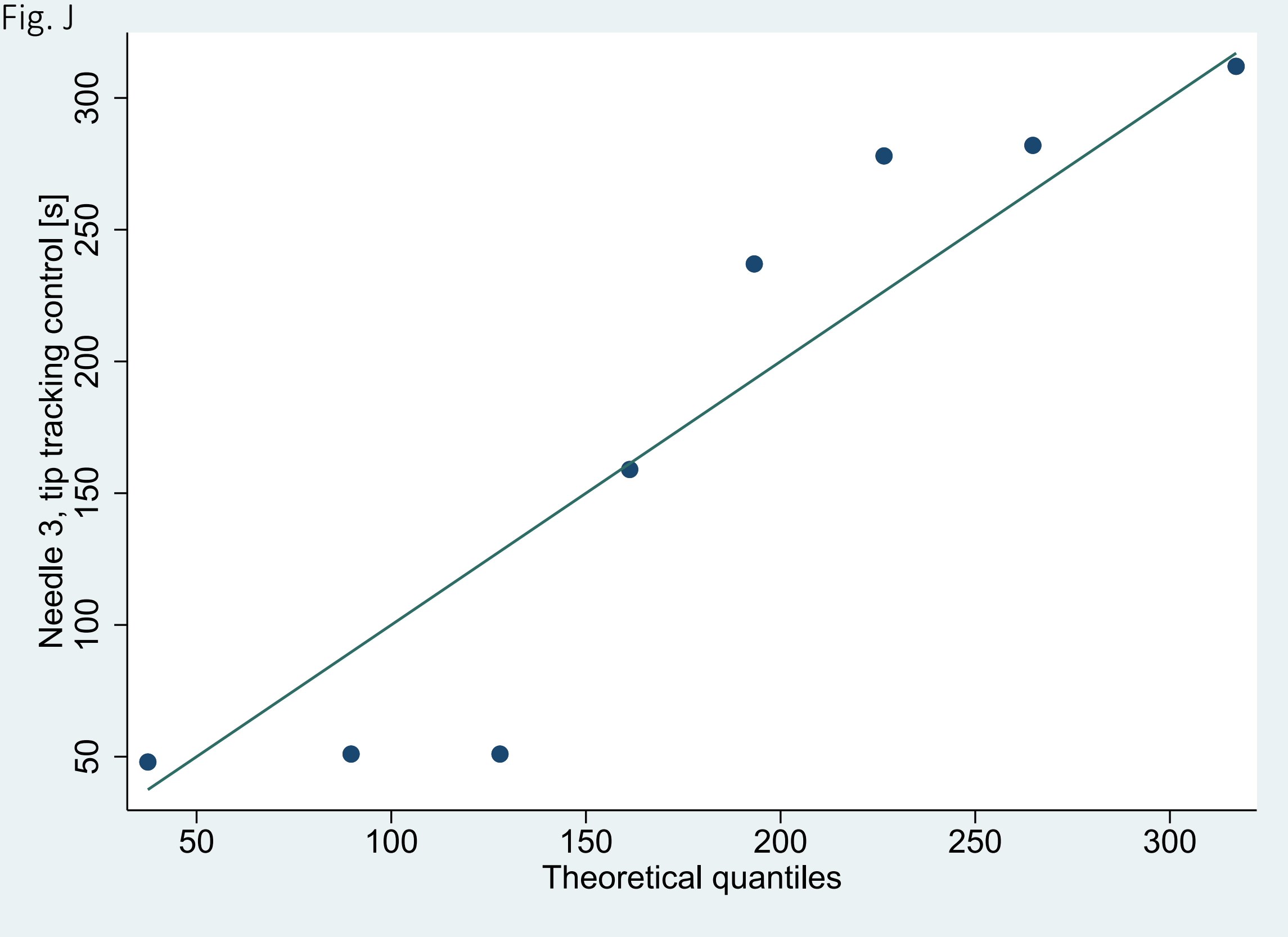


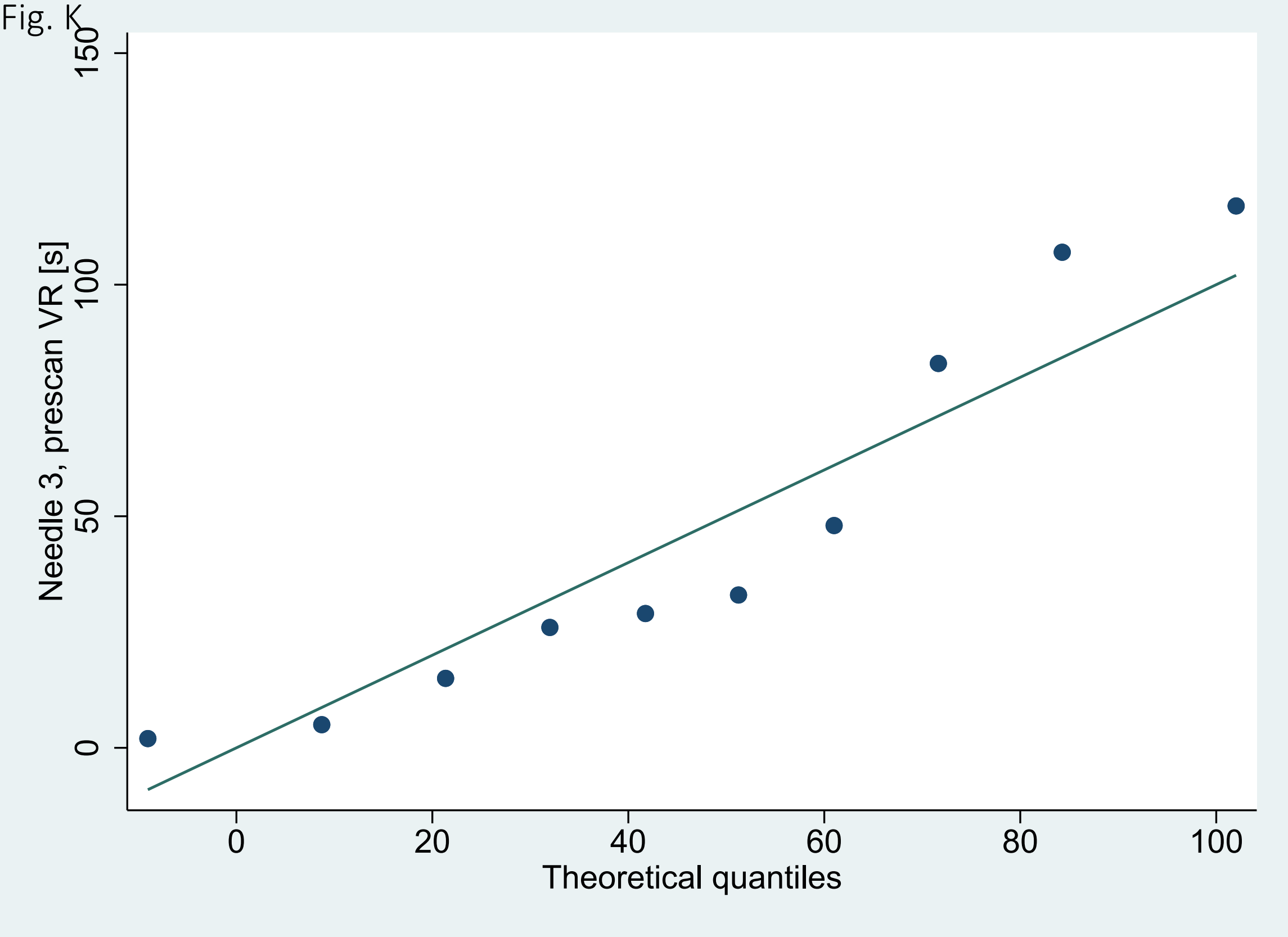

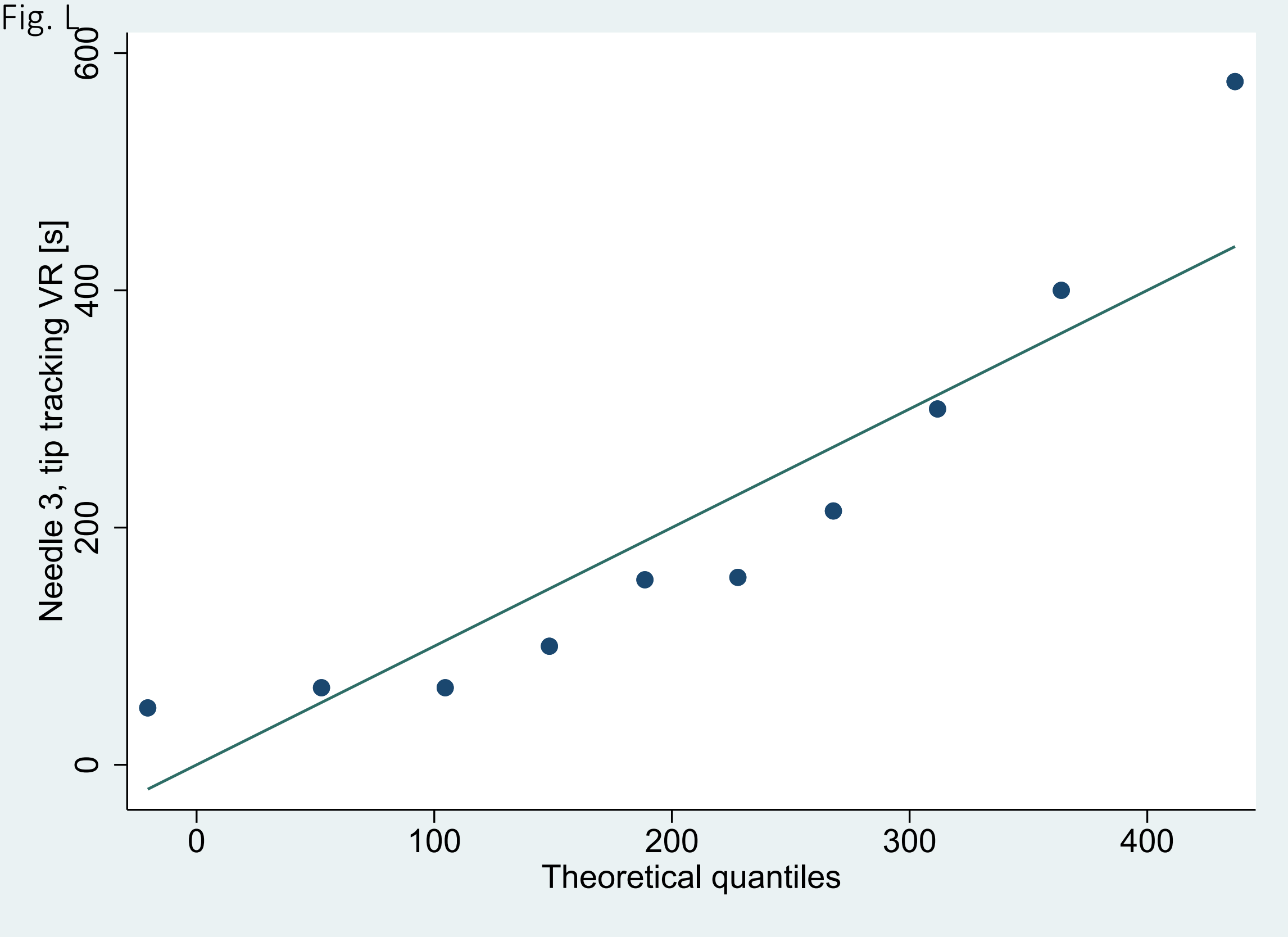

Supplement: Supplemental Digital Content [file medi-100-e26394-s001.docx]
