# Supplementary material for: Teaching ultrasound-guided peripheral venous catheter placement through immersive virtual reality: An explorative pilot study
Source: Medicine (Baltimore). 2021 Jul 9;100(27):e26394. doi: 10.1097/MD.0000000000026394 (PMC8270624; doi:10.1097/MD.0000000000026394)
Supplement: Supplemental Digital Content [file medi-100-e26394-s002.docx]

**Supplemental Digital Content 2: Results for procedure time – normal distribution and two-sample t-test**

| *Parameters* | | *Results for intervention group* | | | *Results for control group* | |
| --- | --- | --- | --- | --- | --- | --- |
| **Shapiro-Wilks test** (> .05 = normal distribution) | | | | | | |
| *Prescan time* | | | | | | |
| Cannula 1 | | <.001 | | | .37 | |
| Cannula 2 | | .17 | | | .90 | |
| Cannula 3 | | .13 | | | .96 | |
| *Tip tracking time* | | | | | | |
| Cannula 1 | | .11 | | | .10 | |
| Cannula 2 | | .512 | | | .54 | |
| Cannula 3 | | .10 | | | .12 | |
| **Two-sample t-test** | | | | | | |
| *Parameter* | *Observations* | *Mean time [s]* | *SE* | *SD* | *95% CI* | *t and p* |
| ***Cannula 1 - prescan*** | | | | | | |
| *Control* | 9 | 48.3 | 4.0 | 11.8 | [39.2 – 57.4] | t = 0.82 **p = .43** |
| *Intervention* | 10 | 38.6 | 10.7 | 33.8 | [14.4 – 62.8] |  |
| *Difference* | - | 9.7 | 11.9 | - | [-15.4 – 34.8] |  |
| ***Cannula 2 - prescan*** | | | | | | |
| *Control* | 9 | 26.6 | 3.8 | 11.5 | [17.8 – 35.4] | t = -0.99 **p = .37** |
| *Intervention* | 10 | 37.0 | 9.4 | 29.7 | [15.8 – 58.2] |  |
| *Difference* | - | -10.4 | 5.3 | - | [-32.7 – 11.8] |  |
| ***Cannula 3 - prescan*** | | | | | | |
| *Control* | 8 | 42.3 | 6.1 | 17.3 | [27.8 – 56.7] | t = -0.27 **p = .79** |
| *Intervention* | 10 | 46.5 | 13.2 | 41.6 | [16.8 – 76.3] |  |
| *Difference* | - | -4.3 | 7.6 | - | [-37.7 – 29.2] |  |
| ***Cannula 1 – tip tracking*** | | | | | | |
| *Control* | 9 | 195.4 | 33.0 | 99.0 | [119.4 – 271.5] | t = 0.21 **p = .11** |
| *Intervention* | 10 | 142.3 | 25.3 | 80.1 | [85.0 – 199.6] |  |
| *Difference* | - | 53.1 | 41.1 | - | [-33.6 – 139.9] |  |
| ***Cannula 2 – tip tracking*** | | | | | | |
| *Control* | 9 | 267.4 | 61.5 | 184.4 | [125.7 – 409.2] | t = 0.87 **p = .40** |
| *Intervention* | 10 | 206.0 | 37.7 | 119.2 | [120.8 – 291.2] |  |
| *Difference* | - | 61.4 | 70.5 | - | [-87.2 – 210.1] |  |
| ***Cannula 3 – tip tracking*** | | | | | | |
| *Control* | 8 | 177.3 | 40.5 | 114.5 | [81.5 – 273.0] | t = -0.44 **p = .67** |
| *Intervention* | 10 | 208.2 | 54.2 | 171.3 | [85.6 – 330.8] |  |
| *Difference* | - | -31.0 | 70.8 | - | [-180.9 – 119.0] |  |
